# Multiphase Actuation of AC Electrothermal Micropump

**DOI:** 10.3390/mi14040758

**Published:** 2023-03-29

**Authors:** Stirling Cenaiko, Thomas Lijnse, Colin Dalton

**Affiliations:** 1 Biomedical Engineering Department, University of Calgary, Calgary, AB T2N 1N4, Canada; 2 Electrical and Software Engineering Department, University of Calgary, Calgary, AB T2N 1N4, Canada

**Keywords:** electrothermal, simulation, multiphase, micropump, electrokinetic

## Abstract

Electrothermal micropumps apply an AC electric field to a conductive fluid within the range of 10 kHz–1 MHz to generate fluid flow. In this frequency range, coulombic forces dominate fluid interactions over opposing dielectric forces, resulting in high flow rates (~50–100 μm/s). To date, the electrothermal effect—using asymmetrical electrodes—has been tested only with single-phase and 2-phase actuation, while dielectrophoretic micropumps have shown improved flow rates with 3- and 4-phase actuation. Simulating muti-phase signals in COMSOL Multiphysics requires additional modules and a more involved implementation to accurately represent the electrothermal effect in a micropump. Here, we report detailed simulations of the electrothermal effect under multi-phase conditions, including single-phase, 2-phase, 3-phase and 4-phase actuation patterns. These computational models indicate that 2-phase actuation leads to the highest flow rate, with 3-phase resulting in a 5% reduced flow rate and 4-phase resulting in an 11% reduced flow rate compared to 2-phase. With these modifications to the simulation, various actuation patterns can later be tested in COMSOL for a range of electrokinetic techniques.

## 1. Introduction

Developing methods of miniaturization and automation of biomedical devices is an important step in further advancing point-of-care (POC) medicine. POC devices, such as lab-on-a-chip (LOC), enable testing to be performed at the site of patient care without samples being sent to a lab with specialized equipment, thereby minimizing delays in treatment. In addition, these devices have lower power requirements, decreased analysis time, increased sensitivity with smaller sample sizes and minimize waste in the process. One of the challenges experienced in developing LOC devices for more complicated lab tests that require multiple steps—such as enzyme-linked immunosorbent assay (ELISA) or polymerase chain reaction (PCR) tests—is in developing adequate micromanipulation techniques. The methods currently used are typically syringe pumps and mechanical micropumps [1]. The disadvantages of these are that they require external tubing to attach the micropump and cannot be fully integrated with the microdevice and syringe pumps are relatively large external items of equipment that typically require mains power to operate. An alternative to these methods includes electrokinetic methods. Electrokinetics includes a group of methods which use electrodes in solution to either sort, mix or pump fluids via electrical forces.

The AC electrothermal (ACET) effect is particularly of interest for this research because of its efficacy in manipulating fluids with a high conductivity, which includes many biofluids (e.g., blood, urine and saliva), biological buffers and pharmaceuticals. ACET devices have been shown to reduce testing times to under 60 s and increase sensitivity for organ-on-a-chip, particle detection and wearable microfluidic devices [1,2,3,4,5,6,7,8,9,10]. Current attempts to increase the flow rates and/or improve mixing in ACET devices consist of various complex electrode and channel geometries to improve the flow profile [11,12,13]. Since these are very difficult or impossible to reliably fabricate with current technologies, there is a need to develop more efficient methods of improving flow rates, particularly with scaling device fabrication in mind. In this paper, multi-phase electrode actuation is numerically studied using COMSOL multi-physics simulations to investigate methods to increase the flow rates in ACET devices.

Computational modeling allows for complex ideas to be tested prior to expending resources on potentially complex and costly fabrication and experimental testing. Concepts that have been investigated by simulation for ACET micropumps include micro-grooved structures embedded in the channel wall to alter the electric field patterns in the fluid and achieve a higher flow rate [11], and different substrate geometries, such as a trapezoidal channel wall and cylindrical channels, where electrodes are patterned on the inside of a tube-like structure [12,13]. One simulated ACET structure required angled electrodes, which would need a multi-layered photolithography process, in which a silicon substrate can be etched, and electrodes patterned on top in a particular way to cultivate the angled electrodes [14,15]. Currently it is very complicated and time consuming to fabricate devices using multi-layered photolithography, especially at sub-100 μm scales. These techniques can potentially be scaled and automated but require significant resources to fabricate and test throughout development. Here, simulations of multiphase configurations are conducted, as fabricating 3- and 4-phase devices would require a complicated multi-layer photolithography fabrication process [14].

In this work, ACET micropumps are simulated with single, 2-, 3- and 4-phase actuation of the electrodes to potentially improve flow rates. Two-phase actuation of ACET micropumps have previously been reported, showing a marked improvement in flow rates of up to 50% [16]. These papers used a DC model and other multi-phase configurations were not previously researched for ACET devices. Multi-phase signals are well documented in the case of traveling-wave dielectrophoresis (twDEP), a particle force that takes advantage of electrorotation forces imposed on the particle by having each electrode out-of-phase with subsequent electrodes [17,18,19,20]. There has also been research in the fields of interfacial electrohydrodynamic and electroosmotic devices that have utilized some form of spatially varying electric field [21,22]. This research investigates instead applying multi-phase signals to ACET devices to increase the electric field strength in the fluid and therefore increasing flow rates as well. The specific methods by which these simulations are performed in COMSOL are discussed in detail.

## 2. Materials and Methods

### 2.1. ACET Theory and Relevant Equations

By establishing an AC electric field within a conductive fluid, a temperature gradient is produced via joule heating, resulting in electrothermal fluid flow. Joule heating can be described by the following energy balance equation, where the left-hand side represents joule heating and the right-hand side is the heat diffusion term [23,24]:

(1) $$\sigma {\left|E\right|}^{2}=-k\nabla^{2}T$$

where σ is the conductivity of the fluid, E is the electric field, k is the thermal conductivity and T is the temperature. The viscous dissipation term is neglected in Equation (1) because this ends up being 10^−10^ times smaller than joule heating. The convective heat term is also ignored since diffusion largely dominates heat transfer, and at sufficiently high frequencies used for electrothermal flow, the equation can be simplified to steady-state [24]. Occasionally, the left-hand term of Equation (1) is represented as 0.5$\sigma {\left|E_{peak}\right|}^{2}$ to account for the root-mean-square time-averaging of the electric field [17]. This temperature gradient causes gradients to form in the electrical properties of the fluid, including the permittivity and conductivity. This varying permittivity and conductivity create a charge density as follows [23]:

(2) $$\rho_{E}=\nabla \cdot \left(\varepsilon E\right)$$

(3) $$\frac{\partial \rho_{E}}{\partial t}+\nabla \cdot \left(\sigma E\right)=0$$

where $\rho_{E}$ is the charge density, ε is the permittivity of the fluid and t is time. By combining Equations (2) and (3), the charge density can be calculated as [23]:

(4) $$\rho_{E}=\frac{\sigma \varepsilon }{\sigma +i\omega \varepsilon }\left(\alpha -\beta \right)\left(\nabla T\cdot E\right)$$

(5) $$\alpha =\frac{1}{\varepsilon }\left(\frac{\partial \varepsilon }{\partial T}\right)$$

(6) $$\beta =\frac{1}{\sigma }\left(\frac{\partial \sigma }{\partial T}\right)$$

where ω is the angular frequency of the applied signal and α and β are estimated as −0.4% K^−1^ and 2% K^−1^, respectively, for aqueous solutions at around 293 K [23]. The electrothermal force, $F_{et}$, is derived from the coulombic and dielectric forces acting on the charge density, which can be represented as [17,23]:

(7) $$F_{et}=\rho_{E}E-0.5E^{2}\nabla \varepsilon$$

The first term in Equation (7) is the coulombic force, while the second term is the dielectric force. The time averaged ACET force equation is as follows [7,9,10,23,24]:

(8) $$F_{et}=0.5\frac{\varepsilon \left(\alpha -\beta \right)}{1+{\left(\omega \tau \right)}^{2}}\left(\nabla T\cdot E\right)E^{*}-0.25\varepsilon \alpha {\left|E\right|}^{2}\nabla T$$

where $E^{*}$ is the conjugate of the electric field and $\tau =\frac{\varepsilon }{\sigma }$ is the charge relaxation time of the fluid. At low frequencies, the coulombic force dominates, while at high frequencies, the dielectric force dominates fluid flow [17,23]. The frequency at which this change occurs is determined by the crossover frequency, which is about 200 MHz for a conductive solution at 1 S/m and far beyond what is used for ACET flow [23]. Although the electric field is oscillating with time, this equation shows that the absolute value of the electric field, which remains constant over time regardless of phase, is used to calculate the electrothermal force. Therefore, this equation will hold true for the multiphase cases discussed here.

Flow rates in COMSOL are established by calculating the Navier–Stokes equations. Here, the steady-state model is used to model the flow rates when the fluid reaches equilibrium. As it was previously shown that the electrothermal force can be time-averaged, the steady-state model applies to the simulation of applied forces. The low Reynolds number approximation is also used, because in these scenarios, the Reynolds number is typically below 0.1. For an incompressible, Newtonian fluid with a low Reynolds number, the steady-state Navier–Stokes equation is [24,25]:

(9) $$\nabla p=\eta \nabla^{2}u+F_{et}$$

where p is pressure, u is velocity and η is the dynamic viscosity. Using Equations (1), (8) and (9), the velocity of fluid due to electrothermal forces can be solved for directly.

### 2.2. COMSOL Simulation

COMSOL is a multiphysics simulation software that includes a wide variety of physics simulations to choose from and allows for easy integration between different modules. Current simulations for ACET micropump have used COMSOL’s electrostatics module and stationary solver to model ACET forces, and to date, have not accounted for AC signals. This essentially treated the problem as an effective DC voltage equivalent to the RMS voltage of a time-varying AC voltage, which only allowed for modeling either single or two-phase (using +V at one electrode and −V at the other) signals [16]. Here, the model is adapted to allow for modeling other actuation patterns in COMSOL.

**Figure 1:**
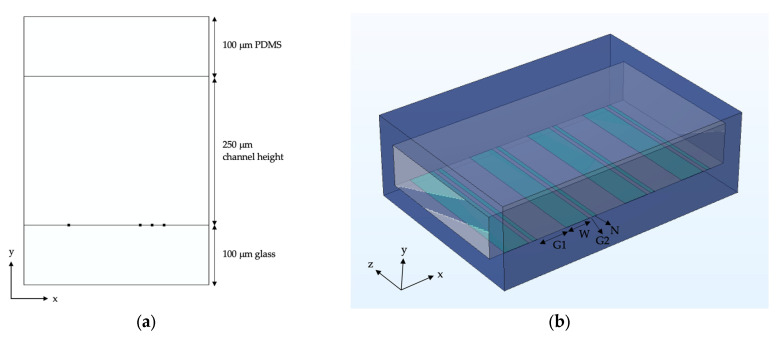
ACET micropump geometry used for COMSOL simulations. (**a**) depicts the cross-sectional geometry of the channel, with the square dots representing the electrode locations at the bottom of the channel and (**b**) shows a 3D model of the channel with electrode dimension definitions for gap 1 (G1), wide electrode (W), gap 2 (G2) and the narrow electrode (N). The x-y-z directions are added for reference in (**a**,**b**), where the z-direction points into the page in (**a**).

Simulations are performed using the Electrical Circuits, Electric Currents, Heat Transfer, Laminar Flow and Electromagnetic Heating modules. Here, each section is discussed in terms of implementation to allow for multi-phase signals. The geometry of the channel is modeled in 2D with dimensions shown in Figure 1. In these simulations, the electrode geometry is as follows: G1 = 150 μm, W = 120 μm, G2 = 20 μm and N = 20 μm, which is in accordance with previous electrode optimization work [26]. Another optimized geometry is also shown to verify the model, with dimensions G1 = 30 μm, W = 15 μm, G2 = 5 μm and N = 1.5 μm [26].

The top of the channel is modeled as polydimethylsiloxane (PDMS) and the bottom of the channel is glass to reflect the fabrication process for current ACET arrays [27]. For these simulations, the fluid is modeled as phosphate buffered saline (PBS) solution with the fluid properties listed in Table 1. The narrow electrodes are set to the desired AC voltage, while the wide electrodes are set to ground potential. The 2D simulations result in about a 18% higher flow rate than 3D simulations for a 750 μm wide channel (into the page), however, the results are relevant for showing trends in data while using significantly less computational resources. An extra-fine mesh was chosen for all simulations since there is only a 0.16% difference in flow rate from extra-fine (17,092 elements) to extremely-fine (29,786 elements) mesh sizes. As before, using a lower mesh resolution significantly reduces computational resources. Flow rates for simulations are given by a line-average of the x-component of velocity at the boundary.

First, the Electrical Circuits and Electric Currents modules are discussed, as they are largely intertwined. Previous work has used the electric currents module alone to simulate ACET flow [28]. Here, the Electrical Circuits module is used to build a circuit for the AC voltage to be applied to the narrow electrodes. This is executed by using a voltage source, where the voltage and phase angle can be specified, in series with ground and connecting this to the External I vs. U port. The port allows the circuit to connect to the model in Electric Currents. The external port is then applied to the terminal in the Electric Currents module to apply the voltage to an electrode. In the Electric Currents module, only the fluid is simulated and the electrically-insulative boundary condition includes all the glass and PDMS walls. The Electric Currents module also uses the periodic condition, as do all remaining modules, to signify that this is one unit in a repeating array.

From here, the Heat Transfer in Solids and Fluids and Electromagnetic Heating modules are implemented. The Heat Transfer defines the solids as the glass and PDMS regions and the channel as the fluid. For the fluid, the velocity is set to the velocity from the Laminar Flow module, where u and v represent the x- and y-components of velocity, respectively. The top of the PDMS and bottom of the glass are modeled as being at 20 °C to represent that the channel exterior is exposed to ambient air at room temperature. To implement the Electromagnetic Heating module to account for joule heating in the fluid, the module is selected from Multiphysics and the electromagnetic component is set to the Electric Currents module.

Finally, the Laminar Flow module is developed for the fluid region. The walls of the glass and PDMS are given a no-slip condition and there is a pressure point constraint added to the bottom right of the channel to give a reference for pressure calculations. A full representation of boundary conditions is provided in previous work [29]. The ACET force from Equation (8) is added as a volume force. However, the equation is rewritten to account for both the real and imaginary components of the electric field, since this appears to be ignored when using the realdot() operator in COMSOL. Equation (10) represents the final force equation input into the simulation for the x-component of the force, while the y-component would follow a similar pattern:

(10) $$F_{et,x}=0.5\frac{\varepsilon \left(\alpha -\beta \right)}{1+{\left(\omega \tau \right)}^{2}}\left[\left(\frac{\partial T}{\partial x}\cdot Re\left(E_{x}\right)+\frac{\partial T}{\partial y}\cdot Re\left(E_{y}\right)\right)Re\left(E_{x}\right)+\left(\frac{\partial T}{\partial x}\cdot Im\left(E_{x}\right)+\frac{\partial T}{\partial y}\cdot Im\left(E_{y}\right)\right)Im\left(E_{x}\right)\right]\;-0.25\varepsilon \alpha \left(Re{\left(E_{x}\right)}^{2}+Re{\left(E_{y}\right)}^{2}+Im{\left(E_{x}\right)}^{2}+Im{\left(E_{y}\right)}^{2}\right)\frac{\partial T}{\partial x}$$

Once the model and mesh are built in the simulation, the frequency-stationary solver is chosen and run at an angular frequency of $\left(2\pi \right)100$ kHz, which is a typical frequency used in ACET devices [27,28,29].

## 3. Results

To validate the new/improved model, simulations are first compared with previous simulations in COMSOL that used the electrostatics module. These results show a 0.0% difference between the new simulation method/model and previous COMSOL models. The new model is also compared with literature from a previously optimized model, with an electrode geometry of 30/15/5/1.5 μm [27]. Compared to the published article, the model described here resulted in only a 7.8% difference in flow rates, which could be due to different versions in COMSOL. The published model geometry (30/15/5/1.5) shows a 305% increase in flow rates compared to the geometry used in these simulations, however, this geometry requires more precise fabrication methods to yield 1.5 μm feature sizes, so the current geometry (150/120/20/20) is kept to provide realistic flow rates from practically realizable devices. In addition, these COMSOL simulations closely follow experimental flowrates yielded from bead tracking measurements, with a simulated flowrate reported here of 102 μm/s compared to a published experimental flowrate of 117 μm/s [30]. The minor difference can be accounted for from the slight variances in device geometry and from using the bead tracking method. Then, the results are compared for models with various phase angle configurations to ensure the imaginary component of the electric field is dealt with appropriately. Since for a single-phase simulation, any phase angle should result in the exact same flow rate, the simulation is tested with 0°, 90° and 120°, all of which yielded the exact same flow rate. For two-phase, the simulations are performed with one electrode set to 0° and the other to 180°, as well as phase angles of 30° and 210°, and these showed the same flow rates as well. This demonstrates that the simulations are treating the real and imaginary components as having an equal effect on flow rates, resulting in the expected flow rates yielded in real-life ACET experiments.

**Figure 2:**
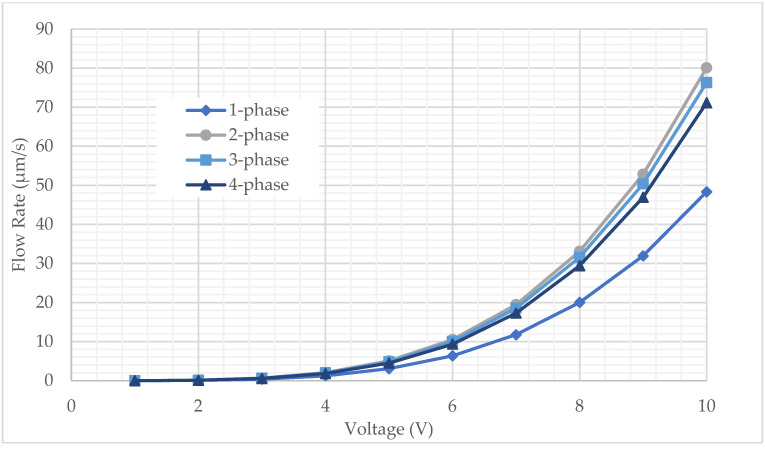
Flow rates for 1-, 2-, 3- and 4-phase configurations at 100 kHz. The voltage given is the peak voltage used in simulations.

**Figure 3:**
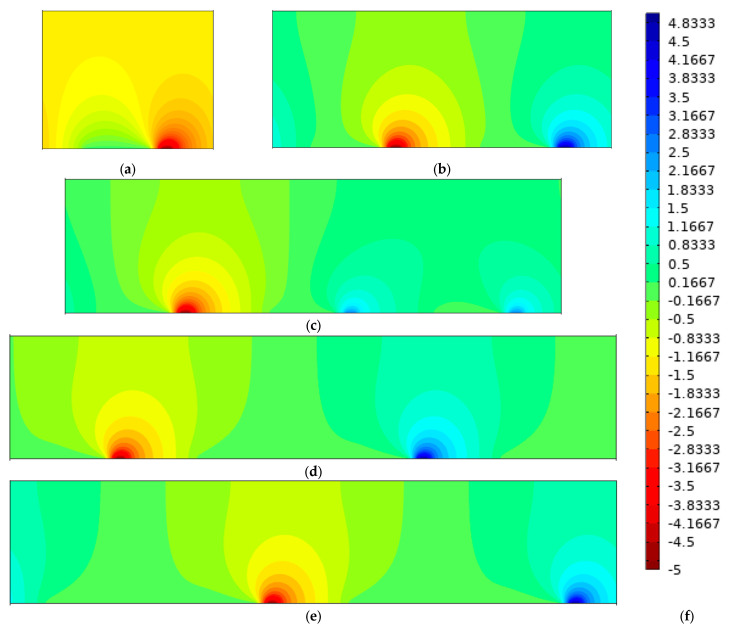
The real component of the voltage profiles is given for (**a**) 1-phase, (**b**) 2-phase (180° phase separation), (**c**) 3-phase (120° phase separation) and (**d**) 4-phase (90° phase separation) configurations at 5 V_peak_, where the far-left electrode is set to a phase angle of 0°. (**e**) shows the imaginary component of the electric field corresponding to the 4-phase configuration in (**d**). (**f**) shows the legend for the voltage contours used in color plots a-e. These simulations are shown for the electrode geometry of 150/120/20/20 μm.

Simulations were then performed for 1-, 2-, 3- and 4-phase configurations of electrodes to compare the performance of multiphase configurations when applied to ACET flow. Results are listed for 5 V_peak_ as a benchmark, since above this fluid can begin to react with the electrodes and damage the device [26]. However, in Figure 2, flow rates are listed up to 10 V_peak_ to provide an idea of flow rates that may be attainable in the future with ACET [31]. Figure 3a–d shows the real component of the voltage throughout the fluid for each phase, where the large electrodes are all set to ground and the small electrodes are set to 5 V_peak_. The complex voltage phasor is equivalent to Vcosθ+jVsinθ, where $j=\sqrt{-1}$ and θ is the phase angle. The narrow electrode furthest to the left is set to 0°, while subsequent electrodes are set to 180° for 2-phase, 120° and 240° for 3-phase and 90°, 180° and 270° for 4-phase. In Figure 3d, the second and fourth narrow electrodes are shown as 0 V because at 90° and 270° the voltage is purely imaginary. Similarly, the electrodes at 0° and 180° have a 0 V imaginary component of the electric field, as shown in Figure 3e.

**Figure 4:**
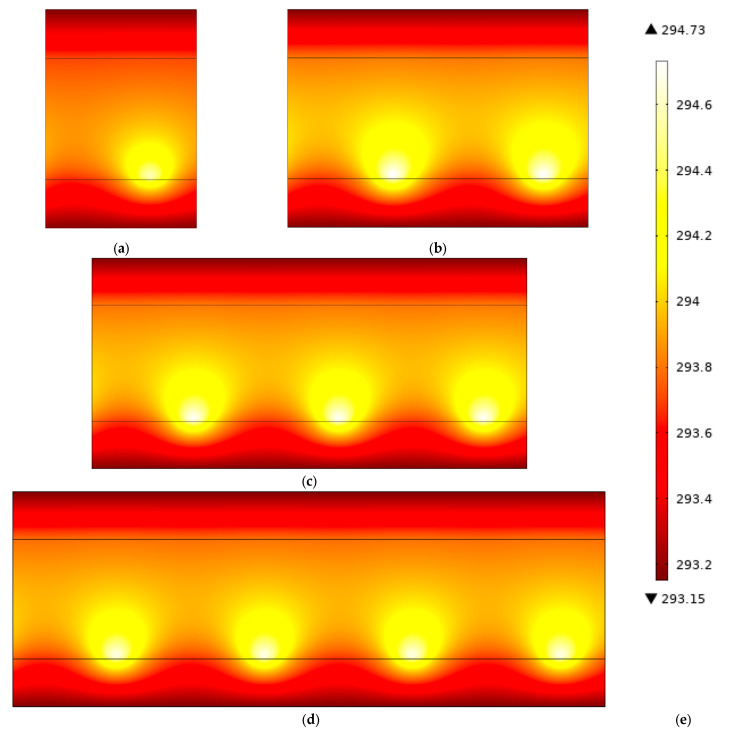
The temperature in an ACET channel, where black lines indicate the fluid-glass boundary, given for (**a**) 1-phase, (**b**) 2-phase (180° phase separation), (**c**) 3-phase (120° phase separation) and (**d**) 4-phase (90° phase separation) configurations at 5 V_peak_, where the far-left electrode is set to a phase angle of 0°. (**e**) shows the color legend for the temperature profiles.

**Figure 5:**
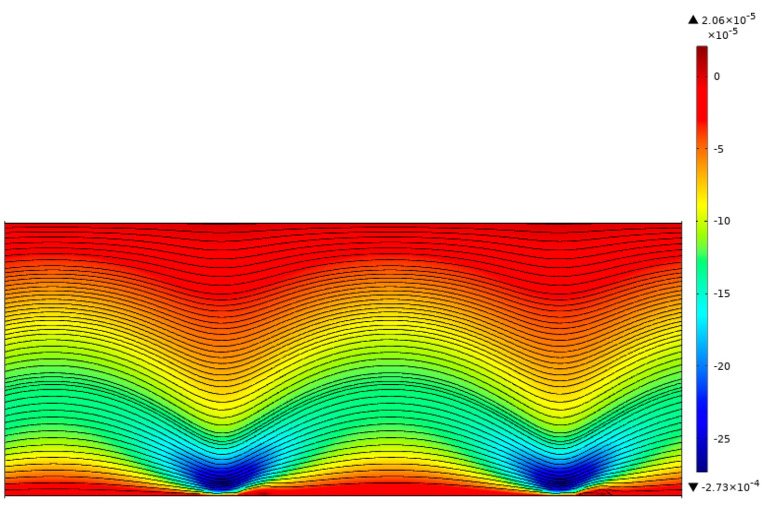
Flow profile for 2-phase ACET micropump at 5 V_peak_.

**Figure 6:**
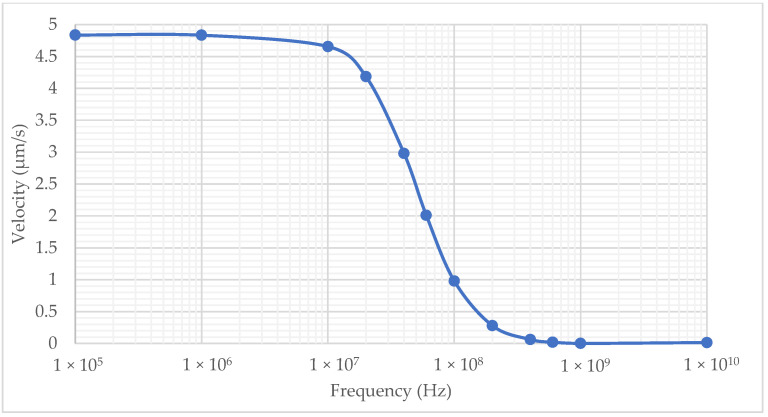
Frequency dependence of ACET flow rates at 5 V_peak_. The ACET flow rate remains relatively constant at lower frequencies but begins to drop off at around 10 MHz as the coulombic force quickly diminishes.

The temperature field is directly related to the electric field, with a higher electric field corresponding to a higher temperature due to joule heating. Temperature profiles are shown in Figure 4. The maximum temperature is located at the region of highest electric field between the electrodes. The maximum temperature for each case at the 5 V_peak_ is as follows: 294.56 K for 1-phase, 294.73 K for 2-phase, 294.70 K for 3-phase and 294.67 K. The temperature is shown to be lower overall for 1-phase as shown in Figure 4 compared to the multiphase cases. These temperatures are all within a safe range for biofluid and cellular transport (<311 K), allowing ACET micropumps to be safely used for medical devices without compromising on results [32]. Flow profiles are very similar in each case, so the flow profile is given for a two-phase micropump in Figure 5. Figure 2 shows the x-component of the velocity for each case vs. the peak voltage applied to the array. This shows that the 2-phase configuration results in the largest ACET velocity, however, 3- and 4-phase also result in an increased velocity as well compared to conventional singular phase ACET micropumps. A frequency sweep is also performed from 100 kHz to 10 GHz in Figure 6 to show the frequency dependence of ACET flow rates. As expected, flow rates tend to remain relatively constant but begin to fall at about 10 MHz as the coulombic force diminishes [16,17].

## 4. Discussion

Here, a simulation is given for multiphase simulations of an ACET micropump to determine what phase configuration is optimal for ACET flow rates. With the previously optimized geometry, a significant increase in the flow rate was observed with this model, which was to be expected, as other optimization attempts have shown that a larger geometry results in significantly decreased flow rates. The results of these simulations show that a 2-phase ACET device can improve flow rates by 65% compared to 1-phase. Flow rates using 3-phase follow closely behind 2-phase, with a relative increase in flow rate of 57% and 4-phase ACET devices have a 47% improvement at 5 V_peak_, compared to 1-phase. This is because the phase difference between electrode pairs results in a higher electric field strength between electrode pairs and therefore a greater ACET force. The average electric field strength between electrode pairs is 4680 V/m for 1-phase, 6890 V/m for 2-phase, 6790 V/m for 3-phase and 6610 V/m for 4-phase. Because the electric field strength directly impacts the temperature gradient and electrothermal force, the electrothermal force follows a similar pattern with the maximum force achieved using a 2-phase configuration and lower calculated forces for 3- and 4-phases. The 2-phase device results in the largest change in voltage over the geometry with an effective 180° phase difference between adjacent small electrodes, leading to the greatest electric field strength. In addition, the multiphase ACET device results in an immeasurably small change in maximum temperature and maintains a low voltage. This method therefore provides a safe way to improve flowrates without risking damage to biofluids due to overheating or undesirable electrochemical reactions.

Although each of these multiphase approaches show a marked improvement to flow rates over single phase, the 2-phase configuration results in the greatest improvement to the flow rate. This paper shows a 65% increase in flow rates by using a 2-phase ACET device. An additional advantage of 2-phase over 3- or 4-phase is that it is easier to fabricate. Using either 3 or 4-phase devices would require that alternating electrodes be connected to one another using a complicated multi-layer fabrication process or by individually connecting each electrode to their respective power sources, requiring significant off-chip cabling. The advantage of 2-phase devices is that they can be fabricated using single-layer photolithography, similar to current 1-phase ACET micropumps, and complex interconnections/vias are not required. The 2-phase devices can be configured so alternating narrow electrodes are connected and a voltage source is applied to the electrodes at 0° and 180°, respectively, while the large electrode remains connected to ground potential. This has the potential to increase the flow rates of ACET devices using an established fabrication procedure and maintaining reasonable operating conditions for biofluids.

## 5. Conclusions

This paper presented and validated a new method to accurately simulate multiphase flow in ACET devices, which was previously not possible. While 3- and 4-phase actuation resulted in an increased velocity compared to conventional singular-phase ACET micropumps, this paper showed that 2-phase actuation provided the maximum flow rate, showing a marked 65% increase in flow velocity. This is greater than can be achieved using either 3- or 4-phase configurations and is simpler to implement. Moreover, the 2-phase configuration can be fabricated using conventional methods of single-layer photolithography, allowing 2-phase ACET micropumps to be easily fabricated and used in place of conventional ACET devices. This new method will enable other actuation patterns to be tested in COMSOL, for both ACET and other electrokinetic techniques. Future work includes experimentally testing the results for 2-phase ACET micropumps and optimizing the geometry for multi-phase devices.

## Figures and Tables

**Table 1:** This table provides all the fluid properties for PBS used in the simulations. The electrical conductivity is given for the measured conductivity of PBS during previous experimental ACET work [16].

Property	Symbol	Value
Relative permittivity	$\varepsilon_{r}$	80.2
Electrical conductivity	σ	0.224 S/m
Thermal conductivity	k	0.598 W/(m K)
Density	ρ	999 kg/m^3^
Heat capacity at constant pressure	$C_{P}$	4181 J/(kg K)
Ratio of specific heats	γ	1
Dynamic viscosity	μ	1.05 × 10^−3^ (N s)/m^2^

## Data Availability

Data is contained within the article.
